# Experiences of Patients With Mental Health Issues Having Web-Based Access to Their Records: National Patient Survey

**DOI:** 10.2196/48008

**Published:** 2024-02-02

**Authors:** Jonas Moll, Gunilla Myreteg, Hanife Rexhepi

**Affiliations:** 1 Centre for Empirical Research on Information Systems Örebro University School of Business Örebro University Örebro Sweden; 2 Department of Business Studies Uppsala University Uppsala Sweden; 3 School of Informatics University of Skövde Skövde Sweden

**Keywords:** patient accessible electronic health record, patient portal, patient experiences, mental health, eHealth, national survey, digital mental health, digital health

## Abstract

**Background:**

Sharing mental health notes through patient accessible electronic health records (PAEHRs) is controversial. Many psychiatric organizations and regions in Sweden have resisted the implementation, as clinicians worry about possible harms when patients are reading their notes. Despite the documented benefits of PAEHRs, there is still a lack of knowledge regarding whether patients with mental health issues could reap similar benefits of reading their notes as other patient groups.

**Objective:**

The aim of the study is to examine the use, attitudes, and experiences of patients with mental health issues by reading their notes in the PAEHR and, moreover, whether their experiences differ from other patient groups, and if so, how.

**Methods:**

A national patient survey was conducted with answers from 2587 patients from different patient groups. In total, 504 respondents (19.5%) indicated that they experienced a mental health disease. Answers from this patient group were compared to the answers from all other respondents. Survey questions related to attitudes, information usage, and effects on contacts with care were selected for analysis. Mann-Whitney *U* tests were used to detect groupwise differences.

**Results:**

Patients with mental health issues use PAEHRs for checking that they have received the right care (mean_mental health 2.83, SD_mental health 1.39; mean_others 2.62, SD_others 1.37; *P*=.002) or suspected inaccuracies (mean_mental health 2.55, SD_mental health 1.34; mean_others 2.31, SD_others 1.30; *P*=.001), blocking access for professionals in other specialties (mean_mental health 3.43, SD_mental health 1.46; mean_others 3.04, SD_others 1.42; *P*<.001), and checking which care professionals have accessed their record (mean_mental health 4.28, SD_mental health 1.14; mean_others 4.05, SD_others 1.25; *P*<.001) to a significantly higher degree than other patients. On the other hand, the results show that a significantly lower proportion of patients with mental health issues (mean_mental health 3.38, SD_mental health 1.21; mean_others 3.52, SD_others 1.18; *P*=.02) believe that PAEHRs help them in shared decision-making compared to other patient groups.

**Conclusions:**

Patients with mental health issues who took part in the survey, as a group, express some minor differences in both the use of the PAEHR and their experiences regarding its usefulness, as compared to other patients, as a group. This patient group shows a slightly higher interest in 2 types of use: checking for accuracy of care in the record and blocking access to mental health notes for professionals from other parts of the health care system. Compared to other patient groups, these patients are less likely to experience that the PAEHR is a support in shared decision-making. The study indicates that the benefits of PAEHR on a general level are the same for this patient group as for other patients. The study does not support clinicians’ worry about possible harm to this patient group. Further research is however needed.

## Introduction

Patient accessible electronic health records (PAEHRs) aim to promote patients’ engagement with their care by giving patients direct access to their electronic health records (EHRs) through a national patient portal. Patients in around 20 countries worldwide, including Estonia, the Nordic countries, Australia, the United States, Canada, and England, are now offered web-based access to at least some of their EHRs. In Sweden, the PAEHR called “Journalen” was launched in 2012, when the region of Uppsala offered all citizens 18 years and older of age access to their EHRs through the national patient portal 1177 Vårdguiden. In 2015, Journalen was launched as the national system in Sweden for web-based access to clinical notes, and at the end of 2018, all regions had implemented Journalen. The PAEHR offers the patient access to his or her medical notes, prescribed medications, laboratory results, diagnosis, maternity care records, referrals, and vaccinations. Since health care in Sweden is governed by 21 autonomous regions with their own regulations, there are some regional differences concerning what type of information a patient can access and how soon (immediately or after 2 weeks). All regions offer patients access to visit notes from somatic care and test results.

Despite the documented benefits of PAEHRs [1-3], clinicians have raised concerns that patients could become confused or anxious by what they read [4]. The web-based access to mental health notes is especially controversial. The clinicians’ main argument is that in mental health, the information concerns sensitive topics that can have negative consequences for patients when they access their notes. In the study by Peck et al [5], several clinicians approved of the possibility to exclude patients from access, when they were considered too vulnerable. Different survey studies related to PAEHR and mental health suggest that clinicians worry about possible harms, and many health care professionals anticipate that patients will become confused, get angry, or decompensate when reading their notes [6,7]. Other studies report that patients with mental health can benefit from accessing their notes. Some reported benefits are increased feelings of engagement [8-10], feeling of control over their health, trusting their providers, taking better care of themselves, remembering their care plan, understanding better the rationale for medications, and being more likely to take their medications as prescribed [11,12]. A small number of studies have however found negative consequences for patients with mental health issues because of reading the mental health notes, such as feeling judged, worried, or offended [5,12-14]. A majority of studies [5,9,15] suggest that outpatient patients with mental health issues value reading their notes, that psychiatrists do not experience increased work burden or perceive negative outcomes, and that respectful, accurate mental health notes may enhance patient trust.

In Sweden, region Skåne made mental health notes accessible to adult patients from 2015. Since then, more regions have followed, and as of today, 17 of 21 regions share mental health notes through the PAEHR Journalen [16]. There are no other differences between patients with mental health issues and other patient groups regarding what information types you have access to and when. Thus, patients with mental health issues have the same access to notes from somatic care, test results, and other information types accessible in their region as all other patients. Attitudes among physicians were studied (along with other professional affiliations) before and after the implementation of PAEHR in region Skåne [7]. That study reported that some physicians were more careful with what they documented in the record, as a result of not knowing how the patient might interpret and use the information. Similar results were reported by Dobscha et al [6] and Denneson et al [17], who found that clinicians were less detailed and changed their tone of the notes when they knew that the patient might choose to read the notes. Respondents in the study by Petersson and Erlingsdóttir [7] also indicated a fear of increasing tension between clinicians and the patient, which could manifest itself in threats and acts of violence. In their follow-up study, the professionals rather expressed that there were no changes in patient involvement after the implementation of PAEHRs [18].

Additionally, Denneson et al [17] reported that clinicians expressed concern that access to mental health notes “could damage the therapeutic relationship by exposing a disconnect between the patients’ in-person experience with their clinicians and the documentation they read in their notes.” Mental health notes can, they argue, reveal aspects of the therapeutic process—such as clinical formulations and subjective impressions—which clinicians frequently do not communicate to their patients. Thus, a patient reading his or her notes could cause the patient to misinterpret the clinician’s notation, which could have negative effects on the patient, such as having feelings of being judged or stigmatized [17]. Moreover, patients with mental health issues are also generally considered a vulnerable patient group, which begs the question whether patients with mental health conditions can reap similar benefits of accessing their PAEHRs as other patient groups [19]. This review shows the topic to be controversial, and in practice, many psychiatric organizations resist implementation.

Empirical research is scarce, especially in Sweden. To our knowledge, there is no comparative research on how PAEHRs are perceived among patients with mental health issues as compared to other patient groups. Moreover, Petersson and Erlingsdóttir [18] make a call for empirical research regarding the perspectives of patients with mental health issues toward PAEHRs. The aim of this paper is to examine the use, attitudes, and experiences of patients with mental health issues by reading their notes in the PAEHR and if their experiences differ from other patient groups. More practical knowledge is needed in this area as input to the ongoing debate regarding the possible benefits for patients in accessing their mental health notes through PAEHRs.

## Methods

### Ethical Considerations

The survey, which focused on attitudes toward and experiences of using Journalen, was approved by the regional ethical review board in Uppsala, Sweden (EPN 2017/045). The respondents were informed about the voluntary participation and the aim of the study as well as presented with standard consent that needed to be accepted before the survey could be started. No data were stored unless the respondent chose to submit the answers at the end of the survey. The data were anonymized by representing each respondent with a number. No incentives were offered for participation.

### Study Design

This paper is based on data from an open anonymous self-completion digital national patient survey distributed to users of the Swedish PAEHR system, Journalen, through a link on the login page. Thus, all citizens who logged in to the service during the period that the questionnaire was accessible (June to October 2016) were potential respondents to the voluntary survey.

The survey included 24 questions with a combination of Likert-scale items, multiple-choice items, and free-text alternatives. The questions covered the following themes: attitudes and reactions, access to and usage of information, effects on contact with health care, information content, security and privacy, personal health information, and demographics.

The theme “personal health information” included a question about which diagnosis group the respondents identified himself or herself to belong to. The respondents could choose between the alternatives of cancer, mental health, diabetes, high blood pressure, and others. The diagnoses of cancer, diabetes, and high blood pressure were specified as survey alternatives as they are the most common chronic conditions in Sweden. The alternative to mental health was included in order to address the ongoing debate in Sweden regarding whether psychiatric records should be made available and whether this patient group can benefit from accessing their medical record. Of the respondents, 504 people chose to identify themselves as belonging to the group of patients with mental health issues. This constitutes 19.5% (n=504) of the respondents who answered the survey. Globally, about 1 in every 8 people live with a mental disorder—most commonly, an anxiety or depressive disorder [20]. In Sweden, an official national health survey reported that 16% of respondents experienced severe mental difficulties, but that as many as 71% of respondents experienced feelings of anxiety or worry [21]. It is thus not remarkable or questionable that as many as 19.5% (n=504) of respondents in this study identified themselves to belong to this group of patients.

The full national survey was analyzed and presented by Moll et al [22]. In this study, 7 Likert-scale questions, including several items, related to attitudes, information usage, and contacts with care were selected for further analysis in relation to patients with mental health issues. Questions related to general attitudes and information usage were also picked out in order to shed light on any differences regarding how patients view and use the possibilities that Journalen gives. Finally, questions related to information accuracy, contact with care, and involvement in the care process were selected, since they reflect issues that mental health professionals have raised, as reported by previous studies. This set of selected questions is motivated by the need to develop knowledge that addresses the controversial question regarding access of patients with mental health issues to their records, and the set of questions relate closely to the concerns that were raised by health care professionals. The paper focuses on the answers of patients with mental health issues as a group and compares those to the answers from the other respondents as constituting another group.

During the time that the survey was distributed on the login page of Journalen, only 2 regions (Skåne and Kronoberg) had opened up web-based access to mental health notes. One consequence of this is that some of the patients with mental health issues who answered the survey could not yet access the mental health notes, while others could. Patients who lived in other Swedish regions could still access other types of health information (eg, test results and notes from primary care visits). During the survey period, 154 patients (30.6% of the mental health respondents) belonged to Skåne or Kronoberg and could thus access their mental health notes.

### Analysis

Apart from descriptive analysis, Mann-Whitney *U* tests were used for detecting groupwise differences in answers on the 5-point Likert-scale questions between the group of patients with mental health issues and the group of all other respondents. The same test was used for detecting groupwise differences between mental health respondents from the regions Skåne and Kronoberg, who could read the mental health notes at the time the survey was open, and all other mental health respondents. This extra comparison was performed to investigate if the survey answers were affected by the fact that some of the mental health respondents could not actually access their mental health information but only information related to somatic care. Prior to the analysis, the Likert-scale options strongly agree, agree, neutral, disagree, and strongly disagree were converted to a numerical scale (1=strongly disagree and 5=strongly agree). No free-text questions were analyzed in this study. The SPSS software (version 25; IBM Corp) was used for all calculations.

## Results

### Result Presentation

In the tables in the following subsections, the response options “strongly agree” and “agree” have been combined for readability. For the same reason, “strongly disagree” and “disagree” were also combined. In Multimedia Appendix 1, the results for all response options are provided for completeness. As mentioned, some patients with mental health issues could access their mental health notes in Journalen at the time of the study, while others could not. Mann-Whitney *U* tests were used to check if there were any significant differences between patients with mental health issues who could and could not access their mental health notes. Significant differences were only found for 2 of the survey questions in the study (regarding access to all types of record entries and access to log list). Hence, the vast majority of the results presented here were not affected by the respondents being able to access their mental health notes.

### Demographic Information

Demographic information about the respondents is provided in Table 1 together with a comparison against demographic data from the other group of respondents. Chi-square tests were used to check for significant associations between the compared variables. The group of patients with mental health issues was a bit younger, which was expected based on current statistics [23]. Moreover, there was a female dominance that was however a bit stronger than the statistics would suggest. Additionally, the level of education is lower for the mental health group. No differences were found regarding previous work experience in health care.

**Table 1:** Demographic information for respondents who identified themselves as patients with mental health issues.

Demographic		Mental health, n (%)		Others, n (%)		*P* value	
**Age^a^ (years)**							<.001
	18-25	75 (15)	96 (4.9)				
	26-35	145 (29)	269 (13.8)				
	36-45	109 (21.8)	264 (13.5)				
	46-55	89 (17.8)	366 (18.8)				
	56-65	55 (11)	427 (21.9)				
	>66	27 (5.4)	528 (27.1)				
**Sex^b^**							<.001
	Female	387 (78.3)	1242 (63.9)				
	Male	100 (20.3)	698 (35.9)				
	Others	7 (1.4)	3 (0.2)				
**Works or has worked in health care^c^**							.11
	Yes	226 (45.4)	804 (41.4)				
	No	272 (54.6)	1139 (58.6)				
**Education^d^**							.004
	Research education	11 (2.2)	64 (3.3)				
	Higher education ≥3 years	168 (33.8)	776 (39.7)				
	Higher education <3 years	94 (18.9)	372 (19)				
	High school ≥3 years	112 (22.5)	296 (15.1)				
	High school <3 years	56 (11.3)	192 (9.8)				
	Less than high school	32 (6.5)	127 (6.5)				
	No formal education	11 (2.2)	55 (2.8)				
	Others	13 (2.6)	72 (3.7)				

^a^Patients with mental health issues: n=500; other patients: n=1950.

^b^Patients with mental health issues: n=494; other patients: n=1943.

^c^Patients with mental health issues: n=498; other patients: n=1943.

^d^Patients with mental health issues: n=497; other patients: n=1954.

### General Attitudes

Both patients with mental health issues and all other patients are generally positive toward the Swedish PAEHR system, Journalen (Table 2). The vast majority of the respondents, no matter which of the 2 groups they belong to, believe that access to Journalen is good for them (Q3b), and that web-based access to medical records is generally a good reform (Q3a). The Mann-Whitney *U* tests showed significant differences between the 2 groups for questions Q3a (mean_mental health 4.73, SD_mental health 0.68; mean_others 4.80, SD_others 0.60; *P*=.01) and Q3b (mean_mental health 4.78, SD_mental health 0.64; mean_others 4.86, SD_others 0.51; *P*=.005), indicating a slightly less positive attitude among patients with mental health issues. The difference between the 2 groups is, however, very small, pointing to that the results are not that significant.

Regarding the content within the PAEHR Journalen, the respondents were asked to rate how accurate they believe the content is. This question was split into 2 aspects: whether the information that is found in the record is correct (Q15a) and whether sufficient information was recorded (Q15b; Table 3). Both groups, patients with mental health issues and all other patients, gave a fairly high rating regarding the correctness of information (mean_mental health 3.98, SD_mental health 0.99; mean_others 4.22, SD_others 0.91; *P*<.001) and a lower score regarding the completeness of information (mean_mental health 3.32, SD_mental health 1.34; mean_others 2.85, SD_others 1.40; *P*<.001). The differences between the groups were statistically significant in both cases, indicating that patients with mental health issues were more inclined to think that information was complete but less convinced that the information was correct, as compared to all other patients. This being said, the average rating among patients with mental health issues for the question about completeness was fairly low (close to neutral), while it was higher (close to “agree”) for the question about correctness.

**Table 2:** The results regarding general attitudes toward Journalen from patients with mental health issues and all other patients.

Question	Mean_mental health (SD)	Mean_others (SD)	*P* value
I believe that access to medical records online is generally a good reform	4.73 (0.68)	4.80 (0.60)	.01
I believe that access to “*Journalen*” is good for me	4.78 (0.64)	4.86 (0.51)	.005

**Table 3:** The results regarding information accuracy in Journalen from patients with mental health issues and all other patients.

Question	Mean_mental health (SD)	Mean_others (SD)	*P* value
The content in the record reflects the information I think that health care has about me	3.98 (0.99)	4.22 (0.91)	<.001
There is information about me that is missing in the record which I think should be there and that the staff should know	3.32 (1.34)	2.85 (1.40)	<.001

### Accessing Patient Information

Regarding the respondents’ answers to why a patient uses Journalen (Q4a-h), the results show that the most common reasons for accessing Journalen, among patients with mental health issues, are to get an overview of the medical history and treatment (Q4b), to follow-up what has been said during a health care visit (Q4e), and to become more involved in the care (Q4h). The same holds true for all other survey respondents. The least common reason for access, for both groups, was to get an overview of relatives’ medical history and treatment (Q4c). Results for all these questions are presented in Table 4.

There were 4 reasons for access, where the analysis showed significant differences between patients with mental health issues and the other respondents (Q4a,b,d, and f; also in Table 4; detailed results for all items related to this question (Q4) are presented in Multimedia Appendix 1). Of these 4, to get an overview of medical history and treatment, Q4b got the highest mean score, where patients with mental health issues gave slightly lower ratings (mean_mental health 4.58, SD_mental health 0.78) than other patients (mean_others 4.65, SD_others 0.80; *P*=.001). Still, the results show that patients with mental health issues see this as one of the most important reasons for accessing the PAEHR. On the other hand, compared to the other respondents, patients with mental health issues gave slightly higher, significant, ratings for the following reasons to use: general interest (mean_mental health 3.86, SD_mental health 1.19; mean_others 3.66, SD_others 1.29; *P*=.002), insecurity of whether the care is right (mean_mental health 2.83, SD_mental health 1.39; mean_others 2.62, SD_others 1.37; *P*=.002), and suspicion of inaccuracies (mean_mental health 2.55, SD_mental health 1.34; mean_others 2.31, SD_others 1.30; *P*<.001). The differences are, however, not very large, and none of these 3 were marked as one of the most common reasons for access by any of the groups.

Respondents were also asked to rate items of their importance, in connection to being able to access Journalen (Q5). The respondents in the mental health group rated the following benefits of being able to access Journalen the highest: it makes me feel informed (Q5e), it makes me feel safe (Q5d), and it improves communication between medical staff and me (Q5a). The other respondents’ ratings gave similar results aside from that they rated Q5c (it improves the understanding of the condition) as one of the top 3 benefits instead of Q5d. It is also of interest to note that both groups of respondents gave a very low rating to the item it has no relevance (Q5j), indicating that the respondents generally see clear benefits from accessing Journalen. See Table 5 for detailed results.

**Table 4:** Respondents’ answers to the question “Why do you use Journalen?” by patients with mental health issues and all other patients^a^.

Question	Mean_mental health (SD)	Mean_others (SD)	*P* value
Mostly general interest	3.86 (1.19)	3.66 (1.29)	.002^b^
To get an overview of my medical history and treatment	4.58 (0.78)^c^	4.65 (0.80)^c^	.001^b^
To get an overview of my relatives’ medical history and treatment	2.06 (1.45)	2.21 (1.52)	.05
Because I am not sure if I got the right care	2.83 (1.39)	2.62 (1.37)	.002^b^
To follow up what has been said during a health care visit	4.47 (0.91)^c^	4.39 (0.99)^c^	.10
Because I suspect inaccuracies	2.55 (1.34)	2.31 (1.30)	<.001^b^
To prepare for my health care visit	3.40 (1.35)	3.51 (1.33)	.11
To become more involved in my care	4.21 (1.10)^c^	4.28 (1.02)^c^	.39

^a^The Mann-Whitney *U* test was used for statistical analysis.

^b^Significant *P* values.

^c^The most highly ranked options by mental health respondents and other respondents.

**Table 5:** Respondents’ answers to the question “How important is it for you to be able to access patient information?” by patients with mental health issues and all other patients. Some items related to Q5 are only shown in Multimedia Appendix 1^a^.

Question	Mean_mental health (SD)	Mean_others (SD)	*P* value
It improves communication between medical staff and me	4.21 (0.96)^b^	4.36 (0.92)^b^	<.001^c^
It improves the understanding of the condition	4.18 (1.02)	4.26 (0.98)^b^	.12
It makes me feel safe	4.24 (0.99)^b^	4.22 (0.97)	.44
It makes me feel informed	4.59 (0.76)^b^	4.62 (0.72)^b^	.42
It leads to that I can take care of my health better	3.40 (1.16)	3.54 (1.13)	.02^c^
It leads to that I can take care of my relatives health better	2.39 (1.38)	2.52 (1.36)	.04^c^
It is essential that I am able to actively participate in decisions about my or my relatives’ health	3.31 (1.44)	3.47 (1.41)	.03^c^
It has no relevance	1.49 (0.96)	1.50 (0.93)	.52

^a^The Mann-Whitney *U* test was used for statistical analysis.

^b^The most highly ranked options by mental health respondents and other respondents.

^c^Significant *P* values.

Some significant differences were however found between the groups (also in Table 5). Patients with mental health issues rated the following benefits of Journalen as of lower importance than did the other patients: improves communication with health care (*P*<.001), enables better self-care (*P*=.02), improves the possibility to take better care of relatives (*P*=.04), and enables the essential possibility to participate in health decisions (*P*=.03). None of the stated items in Q5 were rated of higher importance by patients with mental health issues compared to other patients, with a significant difference between the groups. Among the items where significant differences could be identified, only item Q5a about improved communication was one of the most highly rated benefits of accessing Journalen.

Moreover, respondents were asked how important different information types and functions in Journalen are to them (Q17a-r). The 3 functions or information types that the group of patients with mental health issues rated to be of highest importance to have access to were results of tests (Q17d), being able to read all types of record entries (Q17g), and overview of all health care contacts (Q17e). This rating corresponds well to the ratings from the group of other patients. Both respondent groups gave the lowest ratings to the importance of being able to communicate electronically with other patients (Q17o).

There were 18 items included in this survey question. For most of the items, no significant difference could be found between the 2 groups of respondents. The 6 items for which significant differences *could* be identified are presented in Table 6. Patients with mental health issues gave higher ratings to the importance of the following information types or functions: psychiatry records (*P*<.001), all types of medical notes or record entries (*P*=.02), blocking professionals from access to certain information (*P*<.001), and access to the log list (*P*<.001). Of these, the differences were largest regarding the importance of having access to the psychiatry record (mean_mental health 4.47, SD_mental health 1.09 and mean_others 3.65, SD_others 1.38) and the possibility to block other professionals from having access to all patient information (mean_mental health 3.43, SD_mental health 1.46 and mean_others 3.04, SD_others 1.42), which was expected due to the special needs of this group. Furthermore, patients with mental health issues gave slightly lower ratings regarding the importance of the following information types or functions: referral tracking (*P*=.02) and test results (*P*=.01). It is also noticeable that both studied groups of patients gave generally high ratings to most of the information types and functions included in question Q17.

For 2 of the information types or functions listed in the survey (access to all record entries and access to the log list), the responses from patients with mental health issues who could or could not access their mental health records differed significantly. In the case of access to all types of record entries, those who could access their mental health notes gave significantly higher ratings (*P*=.049), and in the case of the log list, this group of respondents gave significantly lower ratings (*P*<.001).

**Table 6:** Respondents’ answers to the question “How important is it for you to have access to the following information which is wholly or partly based on information contained in “Journalen”?” by patients with mental health issues and all other patients. Some items related to Q17 are only shown in Multimedia Appendix 1^a^.

Question	Mean_mental health (SD)	Mean_others (SD)	*P* value
Referral (content and how it is handled in care)	4.50 (0.88)	4.62 (0.74)^b^	.02^c^
Results of tests	4.69 (0.76)^b^	4.78 (0.61)^b^	.01^c^
Overview of all health care contacts	4.59 (0.79)^b^	4.61 (0.77)	.60
Being able to read record entries from psychiatry	4.47 (1.09)	3.65 (1.38)	<.001^c^
Being able to read all types of record entries	4.68 (0.83)^b^	4.64 (0.79)^b^	.02^c^
Ability to communicate electronically with other patients	2.15 (1.36)	2.02 (1.24)	.14
Ability to block certain medical records from access by other medical staff	3.43 (1.46)	3.04 (1.42)	<.001^c^
See which care units and staff groups have been inside “*Journalen*” (see log data)	4.28 (1.14)	4.05 (1.25)	<.001^c^

^a^The Mann-Whitney *U* test was used for statistical analysis.

^b^The most highly ranked options by mental health respondents and other respondents.

^c^Significant *P* values.

### Relationship With Health Care and Patient Involvement

Two of the questions in the survey (Q7 and Q16) covered aspects of the patient’s relationship with health care and his or her involvement in the care process. Regarding possible changes in the patient’s relationship to health care (in general) after using Journalen (Q7a) and to health care professionals (more specific) due to communication about Journalen (Q7b and c) and its content (Q7d), no significant differences could be found between respondents in the 2 groups (Table 7). Here, it is again important to remember that not all of the respondents who answered in the role of patients with mental health issues, as of the date of the survey, had access to his or her psychiatry record. The results however show that all patients, regardless of group, experience at least a moderate positive effect on the relationship with health care. Furthermore, patients (regardless of group) and health care professionals generally do not talk about the possibility for the patient to use Journalen nor do they discuss its content.

Regarding patient involvement in the care process, some significant differences were found between the 2 groups (Table 8). Patients with mental health issues gave significantly lower ratings when it came to Journalen’s potential to support communication with medical staff (*P*=.02) and its potential to enable shared decision-making (*P*=.02). No significant differences were found regarding support for following prescription of treatment (*P*=.053) or support for self-care (*P*=.69). Overall, the results indicate that Journalen had at least a moderate positive effect in the involvement in the care process for patients with mental health issues, and the same holds true for the other respondents as well.

**Table 7:** Respondents’ answers to the question “To what extent do you agree with the following statements regarding your relationship with health care?” by patients with mental health issues and all other patients^a^.

Question	Mean_mental health (SD)	Mean_others (SD)	*P* value
To take part of the patient information via “*Journalen*” has affected the relationship with health care system positively	3.80 (1.11)	3.88 (1.07)	.13
Medical staff has informed me about the possibility to read “*Journalen*”	1.82 (1.29)	1.87 (1.24)	.17
Medical staff has encouraged me to use the “*Journalen*”	1.70 (1.12)	1.72 (1.09)	.40
I discuss the content of “*Journalen*” with medical staff	2.53 (1.49)	2.52 (1.41)	.95

^a^The Mann-Whitney *U* test was used for statistical analysis.

**Table 8:** Respondents’ answers to the question “How important is “Journalen” to make you feel that you are involved in your own care?” by patients with mental health issues and all other patients^a^.

Question	Mean_mental health (SD)	Mean_others (SD)	*P* value
Information in “*Journalen*” has helped me in communication with medical staff	3.57 (1.19)	3.71 (1.13)	.02
Information in “*Journalen*” had a positive impact on the ability to work together with medical staff making decisions about care and treatment	3.38 (1.21)	3.52 (1.18)	.02
Information in “*Journalen*” had a positive impact on the ability to follow the prescription of treatment	3.71 (1.22)	3.83 (1.16)	.05
Information in “*Journalen*” had a positive impact on the ability to take own steps to improve health	3.56 (1.22)	3.60 (1.18)	.69

^a^The Mann-Whitney *U* test was used for statistical analysis.

## Discussion

### Principal Findings

The aim of this study was to, through a national patient survey, investigate the experiences of patients with mental health issues with the Swedish PAEHR Journalen, as well as possible differences between patients with mental health issues and other patients, related to experiences with and attitudes toward the eHealth service. The paper contributes most and foremost to a much-needed knowledge about the effects of Journalen for a specific patient group—patients with mental health issues—several years after the launch of the service. Several important conclusions about aspects that patients with mental health issues value with regard to Journalen were identified in this study, and some interesting differences between the groups of patients with mental health issues and all other patients were brought to light in the comparative analysis. The results also reveal that, in most cases, patients with mental health issues see the same values in Journalen as other patients.

First, and on an overall level, it is clear from the results of the survey that respondents in the mental health group, as well as all other respondents, were positive toward being able to access personal health information in Journalen, and that there are no big differences between patients with mental health issues and other patients. These results are in accordance with earlier research [9,12]. These results are important, as health care professionals have raised concerns that patients with mental health issues in particular would become confused and agitated from reading their PAEHR [6,7]. Moreover, the results reveal that the group of patients with mental health issues is somewhat more critical toward the accuracy of the content compared to other respondents. A possible explanation for why more patients with mental health issues find inaccuracies could be that mental health conditions are more subjective and difficult to quantify and therefore may give rise to disagreements in how they should be described and documented. Since patients with mental health issues find more inaccuracies in the record, and since current research [6,17] has reported that some clinicians change the way they document as a result of patients reading their notes, it is of utmost importance that we open up a discussion regarding how notes could or should be written, and if, how, and when the patients should be involved.

Second, regarding the reasons for using the service as well as what information types were considered to be important to patients, there were no big differences between the 2 respondent groups. The reasons for use that were rated highest among mental health respondents are using Journalen for receiving an overview of one’s treatments, following up on what was said during a health care visit, and becoming more involved in the care process. This should be seen as an important result, since it shows that the reasons for implementing Journalen in the first place are as relevant for patients with mental health issues as they are for all other patient groups represented in the survey. Nevertheless, this patient group has been treated differently during the implementation process in that, for example, mental health notes were excluded in all regions during the first 3 years and were only accessible in 2 regions 6 years after launch. Results from the comparison between mental health respondents who could access their mental health notes and those who could not, show, interestingly enough, that there are no significant differences for any of the results related to the reasons for using Journalen. Hence, this study does not show any indication that access to these particular notes makes a difference in the reasons for using Journalen or the attitudes that patients with mental health issues have toward it.

There were some significant differences indicating specific needs that are related to patients with mental health issues, but most of these differences are still small. Hence, even though there were statistically significant differences between the groups, most of the results do not support that there, in practice, would be any big differences between patients with mental health issues and other patients. Patients with mental health issues gave a somewhat higher rating for possibilities of reviewing which of the health care professionals have read the content in the EHR (access to the log list) and for blocking professionals from other health specialties from accessing all information. They also reported somewhat higher feelings of insecurity regarding having received the right care.

The results showed that some patients might use Journalen because of insecurity about receiving the right care. This can be related to some of the concerns raised by mental health professionals. Both Petersson and Erlingsdóttir [7], from the Swedish perspective, and Dobscha et al [6], from the US perspective, reported the general concern that patients would request changes in the health record both due to found inaccuracies and notes that can be considered sensitive and that the patient might not agree with. These studies, from the perspective of health care professionals, report on health care concerns regarding the consequences of patients’ access to mental health notes. This survey’s results show that patients from this group indeed use Journalen to check whether they have received the right care. The perceived importance of the blocking feature in the system as well as the log list of who has accessed the record points toward an insecurity in who can access the information. This could possibly be due to the sensitive nature of the information related to mental health.

Third, when it comes to communication with health care, no big differences between patients with mental health issues and all other patients could be observed. Respondents with mental health conditions, as well as all other respondents, were generally positive regarding the effects on communication with health care, which is in line with existing research [22], but they gave lower ratings when it comes to communicating with health care professionals about the existence of Journalen. The fact that health care professionals generally do not inform patients about Journalen or encourage them to use the service has also been reported in earlier research but then concerning patients with cancer [24]. A reasonable interpretation of this neglect is that the earlier-mentioned concerns raised by health care professionals function as an obstacle.

Finally, with regard to effects on involvement in care, the results were also similar between the 2 groups. However, patients with mental health issues gave significantly lower ratings to actual effects on the relationship with health care and regarding shared decision-making. These results could possibly be related to the concerns on the effects of the therapeutic alliance that mental health professionals have raised [17]. In contrast to these results, a previous study showed that patients with cancer gave significantly higher ratings than all other patient groups and on all items regarding the effects of involvement in care [24]. Earlier studies, not focusing on specific patient groups [25], have shown that patients’ web-based access to medical records has improved the possibility for patients to engage in shared decision-making, something that Rexhepi et al [24] also showed for patients with cancer in Sweden. Similar studies of patients with mental health issues are very few to date. Petersson and Erlingsdóttir [18] showed that most of their answering professionals did *not* experience a higher patient involvement. This survey indicates that patients with mental health issues, regarding participation in decisions related to their care, do not experience the same positive effect as other patient groups. This conclusion is thus in line with the health professionals’ view [18]. This issue is clearly worthy of additional exploration.

### Limitations

As already mentioned, at the time of the survey, only patients from the regions Skåne and Kronoberg could access mental health notes, since the introduction in 2013. Consequently, some of the patients with mental health issues who answered the survey could not yet access the mental health notes, while others in fact could. All patients with mental health issues could, however, access information on somatic care. An additional Mann-Whitney *U* test was used to compare answers between patients with mental health issues who could and could not access mental health notes. For most of the areas covered in this paper, no significant differences could be found between these 2 groups. In light of this study limitation, when it comes to actual experiences, the study is focusing more on experiences of the PAEHR among patients with mental health issues in general than mental health notes in particular. However, regarding attitudes, the study captures the ideas of patients with mental health issues regarding access to his or her mental health notes. It is also important to recognize that answers were gathered through self-report. A unique patient might have had contact with health care for numerous reasons. We cannot be sure whether there are patients with mental health issues in the “others” group who did not disclose their mental health status in the survey. Moreover, one could discuss how homogenous the mental health group is, given the diversity in both type of diagnosis and severity in symptoms that might be visible within the group. The survey did not capture this diversity.

### Conclusions

The study was based on a national patient survey where 19.5% (n=504) of respondents indicated that they experienced a mental health disease. The objectives of the paper were to examine the use, attitudes, and experiences of patients with mental health issues by reading their notes in the PAEHR and, moreover, whether their experiences differ from other patient groups, and if so, how.

A first conclusion, on an overall level, is that patients with mental health issues are as positive in their attitudes toward the access of personal health information in Journalen as other patient groups. This conclusion agrees with previous research. A second conclusion is that patients with mental health issues use Journalen differently in 2 manners, as compared to other patient groups: they check the record for inaccuracies regarding care and information content and they tend to block access to mental health notes for professionals from other parts of the health care system. These differences in usage were not known from previous research. A third conclusion is that patients with mental health issues have somewhat other experiences from Journalen than other patient groups, in that they are less likely to find it supportive of shared decision-making between themselves and their doctor. This was not known from previous research. A final conclusion is that the clinicians’ worry about possible harm to this patient group does not find support by the current empirical evidence. Patients with mental health issues with access to their mental health notes reported the same positive attitudes toward Journalen as did patients with mental health issues with only access to their somatic health notes.

There are many previous studies on how patients access PAEHR and their attitudes to the introduction of such eHealth services. This study contributes with knowledge, through its comparative research design, on how PAEHRs are perceived by patients with mental health issues as compared to other patients. Further research is however needed in this area. For example, the study contributed with insights regarding different usage patterns. It would be valuable with empirical insights and explanations of why patients with mental health issues are somewhat more critical regarding the accuracy of the information content. From the perspective of professionals, previous research has predicted that patient access to mental health notes will have consequences on what and how the professionals write the notes. This study indicates interesting paths for further investigation of that issue. A practical implication is, however, that professionals do not need to be overly concerned about potential harm to the patients. Patients with mental health issues use Journalen and its information by the same reasons as other patient groups. Patients with mental health issues are as positive to the effects on communication with health care as other patient groups, which is in line with previous research.

Patients with mental health issues are a vulnerable group, where professionals anticipate that patients may get confused, judged, worried, or angry when reading their notes. This study did not find support for that. Other studies have reported benefits from accessing the mental health notes, such as feelings of increased engagement, control over their health, and trust toward the professionals. Finally, this study contributes with the insight that the group of patients with mental health issues finds it less possible to engage in shared decision-making as compared to other patient groups. Further research could help us better understand why we need to know more about the obstacles to patient participation and how Journalen can be used to better address practical issues related to feelings of engagement, control, and trust.

## Appendix

Multimedia Appendix 1 Detailed results from all the survey questions covered in this study.

## Abbreviations

EHR: electronic health record
PAEHR: patient accessible electronic health record
