# Supplementary material for: Experiences of Patients With Mental Health Issues Having Web-Based Access to Their Records: National Patient Survey
Source: JMIR Ment Health. 2024 Feb 2;11:e48008. doi: 10.2196/48008 (PMC10873793; doi:10.2196/48008)
Supplement: Multimedia Appendix 1 [file mental_v11i1e48008_app1.docx]

**Table S1.** The results regarding general attitudes towards *Journalen* from Mental health patients and all other patients respectively.

|  | **Strongly agree** | **Agree** | **Neutral** | **Disagree** | **Strongly disagree** | **Statistic test results** | |
| --- | --- | --- | --- | --- | --- | --- | --- |
| **Q3a^a^** |  |  |  |  |  |  | U=487082  *P*=.006 |
| Mental health | 406   (80.7%) | 75 (14.9%) | 8   (1.6%) | 9   (1.8%) | 5  (1.0%) | Mean=4.73  SD=0.677 |  |
| Others | 1746 (85.7%) | 227 (11.1%) | 30 (1.5%) | 14 (0.7%) | 21 (1.0%) | Mean=4.80  SD=0.597 |  |
| **Q3b^b^** |  |  |  |  |  |  | U=484940  *P*=.005 |
| Mental health | 429 (85.8%) | 50   (10.0%) | 9   (1.8%) | 7 (1.4%) | 5 (1.0%) | Mean=4.78  SD=0.641 |  |
| Others | 1827 (90.1%) | 149 (7.3%) | 30 (1.5%) | 8 (0.4%) | 14 (0.7%) | Mean=4.86  SD=0.508 |  |

^a^I believe that access to medical records online is generally a good reform

^b^I believe that access to "*Journalen*" is good for me

**Table S2.** The results regarding information accuracy in *Journalen* from Mental health patients and all other patients respectively.

|  | **Strongly agree** | **Agree** | **Neutral** | **Disagree** | **Strongly disagree** | **Statistic test results** | |
| --- | --- | --- | --- | --- | --- | --- | --- |
| **Q15a^a^** |  |  |  |  |  |  | U=412681  *P*<.001 |
| Mental health | 156 (31.5%) | 233 (47.0%) | 66   (13.3%) | 21 (4.2%) | 20 (4.0%) | Mean=3.98  SD=0.991 |  |
| Others | 878 (44.8%) | 796 (40.6%) | 177 (9.0%) | 66 (3.4%) | 45 (2.3%) | Mean=4.22  SD=0.911 |  |
| **Q15b^b^** |  |  |  |  |  |  | U=379933  *P*<.001 |
| Mental health | 122 (24.7%) | 117 (23.7%) | 110 (22.3%) | 83   (16.8%) | 61   (12.4%) | Mean=3.32  SD=1.339 |  |
| Others | 306 (16.1%) | 366 (19.3%) | 413 (21.7%) | 366 (19.3%) | 448 (23.6%) | Mean=2.85  SD=1.397 |  |

^a^The content in the record reflects the information I think that health care has about me

^b^There is information about me that is missing in the record which I think should be there and that the staff should know

**Table S3.** Answers to the question “Why do you use *Journalen*?” from Mental health patients and all other patients, respectively. The Mann-Whitney test was used for the statistical analysis.

|  | **Strongly agree** | **Agree** | **Neutral** | **Disagree** | **Strongly disagree** | **Statistical test results** | |
| --- | --- | --- | --- | --- | --- | --- | --- |
| **Q4a^a^** |  |  |  |  |  |  | U=435378 *P*=.002 |
| Mental health | 175 (35.4%) | 184 (37.2%) | 65   (13.2%) | 32   (6.5%) | 38   (7.7%) | Mean=3.86 SD=1.192 |  |
| Others | 628 (32.5%) | 567 (29.3%) | 384 (19.9%) | 152 (7.9%) | 201 (10.4%) | Mean=3.66 SD=1.288 |  |
| **Q4b^b^** |  |  |  |  |  |  | U=467047 *P*=.001 |
| Mental health | 349   (70.1%) | 112 (22.5%) | 23 (4.6%) | 5   (1.0%) | 9   (1.8%) | Mean=4.58  SD=0.784 |  |
| Others | 1564 (77.6%) | 307 (15.2%) | 76 (3.8%) | 24 (1.2%) | 45 (2.2%) | Mean=4.65  SD=0.802 |  |
| **Q4c^c^** |  |  |  |  |  |  | U=440132  *P*=.051 |
| Mental health | 56   (11.5%) | 42   (8.6%) | 65   (13.3%) | 35   (7.2%) | 290   (59.4%) | Mean=2.06 SD=1.451 |  |
| Others | 280 (14.7%) | 162 (8.5%) | 277 (14.6%) | 136 (7.2%) | 1047 (55.0%) | Mean=2.21 SD=1.523 |  |
| **Q4d^d^** |  |  |  |  |  |  | U=435023  *P*=.002 |
| Mental health | 69   (14.0%) | 112 (22.8%) | 102 (20.7%) | 86   (17.5%) | 123 (25.0%) | Mean=2.83  SD=1.393 |  |
| Others | 226 (11.7%) | 325 (16.8%) | 462 (23.9%) | 340 (17.6%) | 582 (30.1%) | Mean=2.62  SD=1.369 |  |
| **Q4e^e^** |  |  |  |  |  |  | U=483715 *P*=.10 |
| Mental health | 324   (64.5%) | 131 (26.1%) | 21   (4.2%) | 11 (2.2%) | 15 (3.0%) | Mean=4.47 SD=0.908 |  |
| Others | 1227 (61.9%) | 539 (26.8%) | 125 (6.2%) | 34 (1.7%) | 85 (4.2%) | Mean=4.39 SD=0.985 |  |
| **Q4f^f^** |  |  |  |  |  |  | U=429227  *P*<.001 |
| Mental health | 52   (10.5%) | 74   (15.0%) | 120 (24.3%) | 95   (19.2%) | 153 (31.0%) | Mean=2.55  SD=1.342 |  |
| Others | 153 (7.9%) | 234 (12.1%) | 414 (21.4%) | 390 (20.1%) | 747 (38.5%) | Mean=2.31  SD=1.304 |  |
| **Q4g^g^** |  |  |  |  |  |  | U=464912 *P*=.11 |
| Mental health | 128 (25.8%) | 137 (27.6%) | 110 (22.1%) | 52   (10.5%) | 70   (14.1%) | Mean=3.40 SD=1.347 |  |
| Others | 547 (27.9%) | 590 (30.1%) | 394 (20.1%) | 169 (8.6%) | 260 (13.3%) | Mean=3.51 SD=1.333 |  |
| **Q4h^h^** |  |  |  |  |  |  | U=480858 *P*=.39 |
| Mental health | 272   (54.5%) | 128 (25.7%) | 59   (11.8%) | 13 (2.6%) | 27 (5.4%) | Mean=4.21 SD=1.101 |  |
| Others | 1100 (55.8%) | 523 (26.5%) | 224 (11.4%) | 44 (2.2%) | 80 (4.1%) | Mean=4.28 SD=1.024 |  |

^a^Mostly general interest

^b^To get an overview of my medical history and treatment

^c^To get an overview of my relatives’ medical history and treatment

^d^Because I am not sure if I got the right care

^e^To follow up what has been said during a health care visit

^f^Because I suspect inaccuracies

^g^To prepare for my health care visit

^h^To become more involved in my care

**Table S4.** Answers to the question “How important is it for you to be able to access patient information?” from Mental health patients and all other patients, respectively. The Mann-Whitney test was used for the statistical analysis.

|  | **Strongly agree** | **Agree** | **Neutral** | **Disagree** | **Strongly disagree** | **Statistical test results** | |
| --- | --- | --- | --- | --- | --- | --- | --- |
| **Q5a^a^** |  |  |  |  |  |  | U=457058  *P*<.001 |
| Mental health | 241   (48.0%) | 167 (33.3%) | 67   (13.3%) | 14 (2.8%) | 13 (2.6%) | Mean=4.21  SD=0.956 |  |
| Others | 1177 (58.2%) | 538 (26.6%) | 213 (10.5%) | 53 (2.6%) | 42 (2.1%) | Mean=4.36  SD=0.921 |  |
| **Q5b^b^** |  |  |  |  |  |  | U=481940 *P*=.26 |
| Mental health | 211 (42.1%) | 146 (29.1%) | 107 (21.4%) | 18 (3.6%) | 19 (3.8%) | Mean=4.02 SD=1.059 |  |
| Others | 871 (43.9%) | 598 (30.1%) | 397 (20.0%) | 67 (3.4%) | 52 (2.6%) | Mean=4.09 SD=1.001 |  |
| **Q5c^c^** |  |  |  |  |  |  | U=481041 *P*=.12 |
| Mental health | 252   (50.0%) | 138 (27.4%) | 80   (15.9%) | 19 (3.8%) | 15 (3.0%) | Mean=4.18 SD=1.024 |  |
| Others | 1053 (52.9%) | 560 (28.1%) | 263 (13.2%) | 64 (3.2%) | 50 (2.5%) | Mean=4.26 SD=0.975 |  |
| **Q5d^d^** |  |  |  |  |  |  | U=490885 *P*=.44 |
| Mental health | 256   (51.1%) | 158 (31.5%) | 56   (11.2%) | 15 (3.0%) | 16 (3.2%) | Mean=4.24 SD=0.986 |  |
| Others | 1005 (50.3%) | 575 (28.8%) | 322 (16.1%) | 47 (2.4%) | 51 (2.6%) | Mean=4.22 SD=0.970 |  |
| **Q5e^e^** |  |  |  |  |  |  | U=493115 *P*=.42 |
| Mental health | 351   (70.1%) | 117 (23.3%) | 21 (4.2%) | 3   (0.6%) | 9   (1.8%) | Mean=4.59 SD=0.760 |  |
| Others | 1441 (71.9%) | 439 (21.9%) | 84 (4.2%) | 17 (0.8%) | 24 (1.2%) | Mean=4.62 SD=0.716 |  |
| **Q5f^f^** |  |  |  |  |  |  | U=459088  *P*=.02 |
| Mental health | 102 (20.6%) | 121 (24.4%) | 192 (38.8%) | 34   (6.9%) | 46   (9.3%) | Mean=3.40  SD=1.162 |  |
| Others | 487 (24.6%) | 497 (25.1%) | 736 (37.1%) | 124 (6.3%) | 138 (7.0%) | Mean=3.54  SD=1.133 |  |
| **Q5g^g^** |  |  |  |  |  |  | U=442674  *P*=.04 |
| Mental health | 52   (10.6%) | 39   (7.9%) | 168 (34.1%) | 21   (4.3%) | 212 (43.1%) | Mean=2.39  SD=1.377 |  |
| Others | 214 (11.2%) | 192 (10.1%) | 674 (35.3%) | 125 (6.5%) | 704 (36.9%) | Mean=2.52  SD=1.364 |  |
| **Q5h^h^** |  |  |  |  |  |  | U=456776  *P*=.03 |
| Mental health | 137 (27.7%) | 104 (21.0%) | 127 (25.7%) | 30 (6.1%) | 97   (19.6%) | Mean=3.31  SD=1.438 |  |
| Others | 624 (31.7%) | 427 (21.7%) | 498 (25.3%) | 92 (4.7%) | 328 (16.7%) | Mean=3.47  SD=1.406 |  |
| **Q5i^i^** |  |  |  |  |  |  | U=465877 *P*=.15 |
| Mental health | 230 (46.4%) | 144 (29.0%) | 68   (13.7%) | 19 (3.8%) | 35   (7.1%) | Mean=4.04 SD=1.179 |  |
| Others | 874 (44.7%) | 507 (25.9%) | 312 (16.0%) | 92 (4.7%) | 170 (8.7%) | Mean=3.93 SD=1.254 |  |
| **Q5j^j^** |  |  |  |  |  |  | U=437778 *P*=0.52 |
| Mental health | 12 (2.5%) | 11 (2.3%) | 56   (11.8%) | 39   (8.2%) | 357   (75.2%) | Mean=1.49 SD=0.964 |  |
| Others | 38 (2.0%) | 36 (1.9%) | 243 (13.0%) | 183 (9.8%) | 1371 (73.3%) | Mean=1.50 SD=0.932 |  |

^a^It improves communication between medical staff and me

^b^It leads to improvements in health and social care

^c^It improves the understanding of the condition

^d^It makes me feel safe

^e^It makes me feel informed

^f^It leads to that I can take care of my health better

^g^It leads to that I can take care of my relatives health better

^h^It is essential that I am able to actively participate in decisions about my or my relatives’ health

^i^For my own documentation

^j^It has no relevance

**Table S5.** Answers to the question “How important is it for you to have access the following information which is wholly or partly based on information contained in “*Journalen*”?” from Mental health patients and all other patients, respectively. The Mann-Whitney test was used for the statistical analysis.

|  | **Strongly agree** | **Agree** | **Neutral** | **Disagree** | **Strongly disagree** | **Statistical test results** | |
| --- | --- | --- | --- | --- | --- | --- | --- |
| **Q17a^a^** |  |  |  |  |  |  | U=460552  *P*=.02 |
| Mental health | 345   (68.9%) | 89   (17.8%) | 50   (10.0%) | 7   (1.4%) | 10 (2.0%) | Mean=4.50  SD=0.880 |  |
| Others | 1429 (73.5%) | 336 (17.3%) | 149 (7.7%) | 11 (0.6%) | 19 (1.0%) | Mean=4.62  SD=0.735 |  |
| **Q17b^b^** |  |  |  |  |  |  | U=487043 *P*=.73 |
| Mental health | 381   (75.7%) | 61   (12.1%) | 44   (8.7%) | 8   (1.6%) | 9   (1.8%) | Mean=4.58 SD=0.854 |  |
| Others | 1483 (76.0%) | 272 (13.9%) | 142 (7.3%) | 24 (1.2%) | 30 (1.5%) | Mean=4.62 SD=0.800 |  |
| **Q17c^c^** |  |  |  |  |  |  | U=469108 *P*=.23 |
| Mental health | 312   (62.4%) | 80   (16.0%) | 84   (16.8%) | 8   (1.6%) | 16 (3.2%) | Mean=4.33 SD=1.019 |  |
| Others | 1258 (65.0%) | 307 (15.9%) | 282 (14.6%) | 33 (1.7%) | 54 (2.8%) | Mean=4.39 SD=0.983 |  |
| **Q17d^d^** |  |  |  |  |  |  | U=463168  *P*=.01 |
| Mental health | 396   (79.7%) | 70   (14.1%) | 18 (3.6%) | 2 (0.4%) | 11 (2.2%) | Mean=4.69  SD=0.761 |  |
| Others | 1653 (84.4%) | 213 (10.9%) | 66 (3.4%) | 9 (0.5%) | 17 (0.9%) | Mean=4.78  SD=0.611 |  |
| **Q17e^e^** |  |  |  |  |  |  | U=479941 *P*=.60 |
| Mental health | 367   (73.3%) | 85   (17.0%) | 35   (7.0%) | 8   (1.6%) | 6   (1.2%) | Mean=4.59 SD=0.791 |  |
| Others | 1435 (74.2%) | 322 (16.6%) | 136 (7.0%) | 17 (0.9%) | 25 (1.3%) | Mean=4.61 SD=0.765 |  |
| **Q17f^f^** |  |  |  |  |  |  | U=303666  *P*<.001 |
| Mental health | 368 (74.2%) | 60   (12.1%) | 29   (5.8%) | 10 (2.0%) | 29   (5.8%) | Mean=4.47  SD=1.091 |  |
| Others | 794 (42.0%) | 176 (9.3%) | 618 (32.7%) | 64 (3.4%) | 238 (12.6%) | Mean=3.65  SD=1.376 |  |
| **Q17g^g^** |  |  |  |  |  |  | U=465081  *P*=.02 |
| Mental health | 413 (82.4%) | 49   (9.8%) | 16   (3.2%) | 12 (2.4%) | 11 (2.2%) | Mean=4.68  SD=0.831 |  |
| Others | 1507 (77.2) | 265 (13.6%) | 125 (6.4%) | 23 (1.2%) | 32 (1.6%) | Mean=4.64  SD=0.792 |  |
| **Q17h^h^** |  |  |  |  |  |  | U=466265 *P*=.26 |
| Mental health | 329   (65.9%) | 81   (16.2%) | 62   (12.4%) | 12 (2.4%) | 15 (3.0%) | Mean=4.40 SD=0.997 |  |
| Others | 1223 (63.6%) | 294 (15.3%) | 308 (16.0%) | 37 (1.9%) | 61 (3.2%) | Mean=4.34 SD=1.021 |  |
| **Q17i^i^** |  |  |  |  |  |  | U=473168 *P*=.93 |
| Mental health | 314   (63.7%) | 82   (16.6%) | 57   (11.6%) | 21 (4.3%) | 19 (3.9%) | Mean=4.32 SD=1.081 |  |
| Others | 1204 (62.6%) | 381 (19.8%) | 238 (12.4%) | 49 (2.5%) | 52 (2.7%) | Mean=4.37 SD=0.979 |  |
| **Q17j^j^** |  |  |  |  |  |  | U=469178 *P*=.53 |
| Mental health | 175 (35.3%) | 89   (17.9%) | 108 (21.8%) | 47   (9.5%) | 77   (15.5%) | Mean=3.48 SD=1.442 |  |
| Others | 609 (31.6%) | 381 (19.8%) | 490 (25.4%) | 172 (8.9%) | 274 (14.2%) | Mean=3.46 SD=1.383 |  |
| **Q17k^k^** |  |  |  |  |  |  | U=462617 *P*=.23 |
| Mental health | 260 (52.3%) | 107 (21.5%) | 92   (18.5%) | 14 (2.8%) | 24 (4.8%) | Mean=4.14 SD=1.113 |  |
| Others | 908 (47.1%) | 526 (27.3%) | 361 (18.7%) | 52 (2.7%) | 77 (4.0%) | Mean=4.11 SD=1,057 |  |
| **Q17l^l^** |  |  |  |  |  |  | U=473777 *P*=.95 |
| Mental health | 171 (34.5%) | 89   (17.9%) | 148 (29.8%) | 28   (5.6%) | 60   (12.1%) | Mean=3.57 SD=1.333 |  |
| Others | 628 (32.8%) | 399 (20.8%) | 556 (29.0%) | 133 (6.9%) | 198 (10.3%) | Mean=3.59 SD=1.288 |  |
|  |  |  |  |  |  |  |  |
|  |  |  |  |  |  |  |  |
|  |  |  |  |  |  |  |  |
|  |  |  |  |  |  |  |  |
| **Q17m^m^** |  |  |  |  |  |  | U=455196 *P*=.21 |
| Mental health | 168 (34.0%) | 113 (22.9%) | 124 (25.1%) | 40   (8.1%) | 49   (9.9%) | Mean=3.63 SD=1.294 |  |
| Others | 568 (29.7%) | 456 (23.9%) | 563 (29.5%) | 146 (7.6%) | 177 (9.3%) | Mean=3.57 SD=1.245 |  |
| **Q17n^n^** |  |  |  |  |  |  | U=457018 *P*=.13 |
| Mental health | 260 (52.4%) | 140 (28.2%) | 52   (10.5%) | 18 (3.6%) | 26   (5.2%) | Mean=4.19 SD=1.101 |  |
| Others | 953 (49.6%) | 507 (26.4%) | 292 (15.2%) | 64 (3.3%) | 106 (5.5%) | Mean=4.11 SD=1.125 |  |
| **Q17o^o^** |  |  |  |  |  |  | U=452489 *P*=.14 |
| Mental health | 51   (10.3%) | 30 (6.0%) | 103 (20.8%) | 71   (14.3%) | 241 (48.6%) | Mean=2.15 SD=1.357 |  |
| Others | 130 (6.8%) | 79 (4.2%) | 460 (24.2%) | 268 (14.1%) | 963 (50.7%) | Mean=2.02 SD=1.237 |  |
| **Q17p^p^** |  |  |  |  |  |  | U=398205  *P*<.001 |
| Mental health | 167 (33.7%) | 93   (18.8%) | 108 (21.8%) | 42   (8.5%) | 86   (17.3%) | Mean=3.43  SD=1.460 |  |
| Others | 415 (21.8%) | 289 (15.1%) | 585 (30.7%) | 192 (10.1%) | 427 (22.4%) | Mean=3.04  SD=1.420 |  |
| **Q17q^q^** |  |  |  |  |  |  | U=425904  *P*<.001 |
| Mental health | 308   (62.0%) | 93   (18.7%) | 54   (10.9%) | 12 (2.4%) | 30   (6.0%) | Mean=4.28  SD=1.136 |  |
| Others | 1006 (52.4%) | 389 (20.3%) | 299 (15.6%) | 67 (3.5%) | 158 (8.2%) | Mean=4.05  SD=1.249 |  |
| **Q17r^r^** |  |  |  |  |  |  | U=431753 *P*=.11 |
| Mental health | 214 (43.8%) | 42   (8.6%) | 137 (28.0%) | 6   (1.2%) | 90 (18.4%) | Mean=3.58 SD=1.501 |  |
| Others | 732 (39.6%) | 178 (9.6%) | 523 (28.3%) | 46 (2.5%) | 369 (20.0%) | Mean=3.46 SD=1.513 |  |

^a^Referral (content and how it is handled in care)

^b^List of all pharmaceuticals

^c^Overview of all vaccinations

^d^Results of tests

^e^Overview of all health care contacts

^f^Being able to read record entries from Mental health

^g^Being able to read all types of record entries

^h^Ability to order and manage medical certificate and other certificates

^i^Ability to point out errors I find in the record

^j^Ability to write own comments to text in “Journalen”

^k^Contribute with information on health, for example by providing health declaration for next visit

^l^Contribute with information of self-testing/monitoring at home

^m^Contribute information about expectations for the health care visit

^n^Ability to contact health care electronically and ask questions about medical record

^o^Ability to communicate electronically with other patients

^p^Ability to block certain medical records from access by other medical staff

^q^See which care units and staff groups have been inside “Journalen” (see log data)

^r^Ability to access information and manage services for my children

**Table S6.** Answers to the question “To what extent do you agree with the following statements regarding your relationship with health care?” from Mental health patients and all other patients, respectively. The Mann-Whitney test was used for the statistical analysis.

|  | **Strongly agree** | **Agree** | **Neutral** | **Disagree** | **Strongly disagree** | **Statistical test results** | |
| --- | --- | --- | --- | --- | --- | --- | --- |
| **Q7a^a^** |  |  |  |  |  |  | U=482708  *P*=.13 |
| Mental health | 159 (31.8%) | 159 (31.8%) | 131 (26.2%) | 23 (4.6%) | 28 (5.6%) | Mean=3.80  SD=1.109 |  |
| Others | 706 (35.0%) | 622 (30.9%) | 525 (26.1%) | 72 (3.6%) | 90 (4.5%) | Mean=3.88  SD=1.069 |  |
| **Q7b^b^** |  |  |  |  |  |  | U=478341  *P*=.17 |
| Mental health | 41   (8.2%) | 28   (5.6%) | 49   (9.8%) | 66   (13.2%) | 316   (63.2%) | Mean=1.82  SD=1.290 |  |
| Others | 126 (6.4%) | 140 (7.1%) | 242 (12.2%) | 309 (15.6%) | 1166 (58.8%) | Mean=1.87  SD=1.244 |  |
| **Q7c^c^** |  |  |  |  |  |  | U=484601  *P*=.40 |
| Mental health | 23 (4.6%) | 18 (3.6%) | 68   (13.6%) | 67   (13.4%) | 324   (64.8%) | Mean=1.70  SD=1.120 |  |
| Others | 72 (3.6%) | 90 (4.5%) | 279 (14.1%) | 308 (15.6%) | 1231 (62.2%) | Mean=1.72  SD=1.092 |  |
| **Q7d^d^** |  |  |  |  |  |  | U=496123  *P*=.95 |
| Mental health | 63   (12.6%) | 100 (20.0%) | 77   (15.4%) | 60   (12.0%) | 200 (40.0%) | Mean=2.53  SD=1.486 |  |
| Others | 192 (9.7%) | 411 (20.7%) | 371 (18.7%) | 269 (13.5%) | 745 (37.5%) | Mean=2.52  SD=1.412 |  |

^a^To take part of the patient information via “Journalen” has affected the relationship with health care system positively

^b^Medical staff has informed me about the possibility to read “*Journalen*”

^c^Medical staff has encouraged me to use the “*Journalen*”

^d^I discuss the content of “*Journalen*” with medical staff

**Table S7.** Answers to the question “How important is “*Journalen*” to make you feel that you are involved in your own care?” from Mental health patients and all other patients, respectively. Mann-Whitney tests were used for the statistical analysis.

|  | **Strongly agree** | **Agree** | **Neutral** | **Disagree** | **Strongly disagree** | **Statistical test results** | |
| --- | --- | --- | --- | --- | --- | --- | --- |
| **Q16a^a^** |  |  |  |  |  |  | U=450799  *P*=.02 |
| Mental health | 126 (25.1%) | 153 (30.5%) | 146 (29.1%) | 31 (6.2%) | 45   (9.0%) | Mean=3.57  SD=1.189 |  |
| Others | 549 (28.5%) | 627 (32.6%) | 521 (27.1%) | 98 (5.1%) | 131 (6.8%) | Mean=3.71  SD=1.134 |  |
| **Q16b^b^** |  |  |  |  |  |  | U=441620  *P*=.02 |
| Mental health | 106 (21.4%) | 123 (24.8%) | 170 (34.3%) | 45   (9.1%) | 51   (10.3%) | Mean=3.38  SD=1.211 |  |
| Others | 465 (24.4%) | 522 (27.4%) | 615 (32.2%) | 141 (7.4%) | 164 (8.6%) | Mean=3.52  SD=1.184 |  |
| **Q16c^c^** |  |  |  |  |  |  | U=451189  *P*=.053 |
| Mental health | 161 (32.5%) | 141 (28.5%) | 122 (24.6%) | 29 (5.9%) | 42   (8.5%) | Mean=3.71  SD=1.219 |  |
| Others | 687 (35.7%) | 571 (29.6%) | 453 (23.5%) | 88 (4.6%) | 128 (6.6%) | Mean=3.83  SD=1.159 |  |
| **Q16d^d^** |  |  |  |  |  |  | U=467089  *P*=.69 |
| Mental health | 129 (26.1%) | 149 (30.1%) | 139 (28.1%) | 27   (5.5%) | 51   (10.3%) | Mean=3.56  SD=1.224 |  |
| Others | 523 (27.4%) | 532 (27.9%) | 586 (30.7%) | 112 (5.9%) | 156 (8.2%) | Mean=3.60  SD=1.181 |  |

^a^Information in “*Journalen*” has helped me in communication with medical staff

^b^Information in “*Journalen*” had a positive impact on the ability to work together with medical staff making decisions about care and treatment

^c^Information in “*Journalen*” had a positive impact on the ability to follow the prescription of treatment

^d^Information in “*Journalen*” had a positive impact on the ability to take own steps to improve health
